# Enzyme-treated orange pomace alters acute glycemic response to orange juice

**DOI:** 10.1038/s41387-019-0091-z

**Published:** 2019-08-28

**Authors:** Yancui Huang, Eunyoung Park, Rebecca Replogle, Thomas Boileau, Jin-E. Shin, Britt M. Burton-Freeman, Indika Edirisinghe

**Affiliations:** 1 Center for Nutrition Research, Department of Food Science and Nutrition/Institute for Food Safety and Health, Illinois Institute of Technology, Chicago, IL USA; 2 PepsiCo R&D, Chicago, IL USA

**Keywords:** Nutrition, Diseases

## Abstract

The goal of the present study was to determine the impact of the addition of enzyme-treated orange pomace to orange juice on postprandial glycemic response. Ten healthy subjects (aged 27.9 ± 7.7 years, body mass index 22.1 ± 1.1 kg m^−2^) participated in a randomized, 2-arm, cross-over clinical trial to test the glycemic response to 100% orange juice (OJ) or 100% orange juice with 5 g of enzyme-treated orange pomace fiber (OPF). Blood samples were collected and glucose and insulin concentrations were measured at fasting (0 min) and every 15 min for 2 h after consuming the study juice products. Analysis of the 2 h incremental area under the curve (iAUC_0–2h_) indicated a significant reduction in blood glucose after ingesting the OPF juice compared to the OJ, *p* = 0.02. Peak glucose concentrations were also lowered after the OPF juice compared to the OJ, *p* < 0.05. No significant difference was observed in insulin responses between treatments, *p* > 0.05. Overall, this study demonstrated that adding 5 g of fiber from orange pomace into a serving of OJ attenuated the postprandial glucose response.

## Introduction

Orange pomace is a byproduct of orange juice (OJ) production and is a rich source of fiber. It contains an edible portion of the fruit that includes segments, broken pulp sacs, and a center core. OJ can contribute to total fruit intake; however, a core criticism of fruit juice is its high sugar and low fiber content. Fiber is an important part of a healthy diet and known to attenuate the glycemic response to foods^1–3^. Fiber’s beneficial effect on postprandial glycemia has been observed in healthy populations and people with diabetes or metabolic syndrome^4–6^.

Orange pomace has been shown to lower postprandial glycemia in individuals with increased cardiovascular disease risk. It has been demonstrated that the addition of 5.5 g of fiber from orange pomace to 100% OJ attenuated the glycemic response at certain time points across 7 h and two high fat meals in an overweight population^7^. A study conducted by Chen et al.^8^ demonstrated that the acute consumption of a beverage consisting of orange pomace (5.48 g fiber) with a breakfast meal significantly decreased postprandial glycemic and insulinemic excursions in overweight men^8^.

In the present study, orange pomace was treated with enzymes to reduce viscosity, to improve formulation attributes, and maximize sensory attributes. It is unknown if the enzymatic treatment would affect previously observed attenuation of postprandial glycemia^7,8^. Furthermore, the pomace fiber has been shown to attenuate glycemic excursions when consumed by an at-risk population (i.e. overweight) but the product has not been tested in healthy population. Therefore, the present study was designed to determine if the addition of 5 g of enzyme-treated orange pomace fiber (OPF) to 100% OJ would attenuate postprandial glucose concentration excursions in a healthy adults compared to 100% OJ without pomace as assessed by the 2-h area under the glucose concentration–time curve and peak glucose concentrations.

## Methods

This study was approved by the Institutional Review Board (IRB) of Illinois Institute of Technology (IIT) in Chicago, IL, USA and registered in ClinicalTrials.gov (NCT02962375). The study was a randomized, 2-arm cross-over design to evaluate the effects of one serving 100% OJ versus 100% OJ with added enzyme-treated OPF on 2-h glucose and insulin responses. Enzyme-treated orange pomace is a proprietary ingredient from PepsiCo Inc. The orange pomace ingredient is composed of the fiber types found in oranges including pectin, cellulose, and hemicellulose. The ingredient is enzymatically treated to reduce viscosity to a palatable level for beverages while retaining fiber content (US patent # WO2017035458 A1).

Based on our preliminary data, 10 subjects would have 80% power (*α* = 0.05) to detect a 30.9% difference between group glucose 2h AUC means. Twelve healthy subjects between the ages of 20 and 45 years were recruited to ensure 10 subjects were available for the final analysis. During the screening visit, inclusion criteria were assessed, including body mass index (BMI) between 20.0 and 24.9 kg m^−2^, weight ≥50 kg and being a nonsmoker. Subjects were excluded from the study if they had fasting blood glucose >5.6 mmol L^−1^, had an eating disorder or were taking medications or who had gastic diseases/gastric bypass or dietary supplements that would interfere with study outcomes.

Qualified subjects were randomized to receive a control or test drink on two separate study visits (3–7 days apart). After evaluating subject’s general health, fasting status (10–12 h) and compliance, a catheter was placed in subjects’ non-dominant arm for multiple blood sampling. After baseline sample collection, subjects consumed their assigned study juice drink based on a randomization sequence provided by the study statistician. Drinks were finished within 10 min. The study treatments included a scale-weighed serving of OJ without pulp or OJ with 5 g of pomace fiber. OPF (250 g) and OJ (252 g) were ingested, which is ~250 mL. The study treatments were matched for total available carbohydrate, but differed by the content of dietary fiber, which mostly was accounted for by the addition of the orange pomace in the test drink (Table 1). Enzymatic colorimetric method (Randox, UK) and immunoturbidimetric (Kamiya Bimedicals, USA) methods were used to analyze plasma glucose and insulin, respectively, using automated clinical analyzer (Randox Daytona, UK). Intra-assay % coefficient variation for glucose and insulin was 1.17% and 3.02%, respectively.

**Table 1:** Study drink nutrient analysis

	OPF amount/serving	OJ amount/serving
Total ingredient weight (g)	250	252
Total carbohydrates (g)	29.7	25.0
Available carbohydrates (g)	24.2	24.2
Moisture (g)	215.9	223.0
Ash (g)	1.5	1.6
Fat (g)	0.2	0.1
Protein (g)	2.6	2.2
Total dietary fiber (g)*	5.5	0.8
Pomace fiber (g)	5.0	0

100% Orange juice with pomace fiber (OPF) and 100% orange juice (OJ) nutrient analysis provided by the sponsor. *Five grams of dietary fiber derived from pomace fiber

Statistical analyses were performed using SAS program (SAS 9.4, SAS Institute Inc., USA). Shapiro–Wilk tests, skewness, and kurtosis were used to assess normality for continuous variables and data conformed to normal distribution patterns. Student’s *t*-test was used to test the period effect and treatment effect. Study visit difference was not observed. Therefore, the paired *t*-test was employed to assess statistically significant differences between treatments. Endpoints included the glucose and insulin incremental area under the curve (iAUC) for 2 h, peak concentrations for glucose and insulin (*C*_max_), and time maximum (*T*_max_) time when *C*_max_ was achieved. A *p*-value of ≤0.05 was considered statistically significant.

## Results

**Fig. 1:**
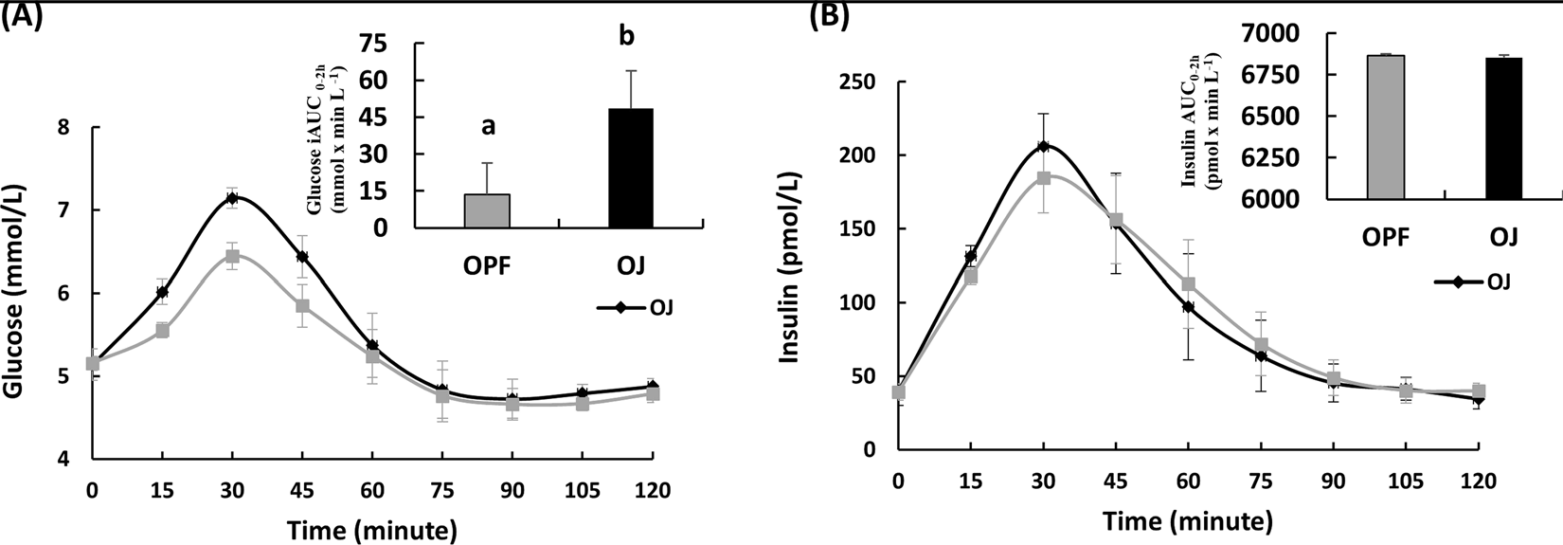
Postprandial glycemic responses. **a** Postprandial plasma glucose concentrations. **b** Postprandial plasma insulin concentrations in response to either 100% orange juice (OJ) or 100% orange juice with pomace fiber (OPF). ^a,b^Overall 2-h postprandial glucose response as estimated by iAUC was significantly lowered after consuming the OPF drink compared to the OJ drink, *p* = 0.02, as indicated by different superscript letters. Overall 2-h postprandial insulin response as estimated by iAUC was not significantly different after consuming the OPF drink compared to the OJ drink, *p* = 0.98. Data shown as mean ± SEM

Ten subjects completed the study (aged 28 ± 8 years, had BMI 22.1 ± 1.1 kg m^−2^ and had fasting blood glucose <5.6 mmol L^−1^). Overall 2-h postprandial glucose responses as estimated by iAUC were significantly lowered after consuming the OPF compared to the OJ (13.5 ± 12.7 vs. 48.5 ± 15.3 mmol min L^−1^, *p* = 0.02, respectively) (Fig. 1a). Glucose *C*_max_ was significantly lowered with the OPF compared to the OJ (6.5 ± 0.8 vs. 7.2 ± 0.9 mmol L^−1^, *p* < 0.001, respectively). No significant effect on the glucose *T*_max_ was observed between the two treatments, although there was a slight delay in the *T*_max_ after the OPF compared to the OJ (33.0 ± 6.3 vs. 31.5 ± 4.7 min, *p* > 0.05).

The treatment effect on insulin responses estimated by iAUC was not significantly different between OPF and OJ treatments (6863.5 ± 1387.6 vs. 6853.3 ± 1221.6 pmol x min L^−1^, *p* = 0.98, Fig. 1b). Insulin *C*_max_ and *T*_max_ also were not significantly different between the treatments (Insulin *C*_max_ 186.8 ± 93.8 vs. 212.5 ± 114.6 pmol L^−1^, *p* = 0.2 and *T*_max_ 34.5 ± 7.2 vs. 30.0 ± 7.1 min, *p* > 0.05).

## Discussion

This acute cross over study indicated that adding 5 g of enzyme-treated OPF to OJ significantly attenuated the postprandial glycemic response in healthy human subjects. The reduction was 3.6-fold for iAUC _(0–2h)_ and 1.1 fold lower for peak glucose. This study result is consistent with previous investigations demonstrating reduced postprandial glycemia with addition of OPF to juice with a meal^7,8^. Although the OPF in the present study was enzyme-treated to reduce the viscosity, the glucose-lowering effect appears to have been maintained. Dong et al.^7^ suggested the glucose-lowering effect could be due to the effect of the pomace fiber reducing glucose availability for absorption through delayed stomach empting, binding glucose, and/or increasing the passage of food through the gastrointestinal tract^7^. Although the mechanism of action cannot be determined, data in this study suggest that less glucose was available for absorption. Reducing glucose absorption is associated with a reduced insulin response^9^. However, in the present study, we did not observe any significant effect on postprandial insulin concentrations (iAUC_0–2h_) or *C*_max_, although a trend in decreased *C*_max_ was observe with OPF. One possible explanation is that the study was statistically powered to observe glucose iAUC_0–2h_ or *C*_max._ On the other hand, subjects in the present study were healthy with normal fasting glucose/insulin levels. Therefore, the administered glucose load in both treatments may not have been sufficient to observe any effect on insulin concentrations. However, subjects with hyperinsulinemia and impaired glucose tolerance have demonstrated detectable changes in both postprandial glucose and insulin after relatively low carbohydrate meals with different fiber composition^10^.

In addition to a fiber-specific effect on post-prandial glucose concentration, OJ contains flavonoids, particularly hesperidin, which has glucose lowering effects^7^. Administration of hesperidin for 30 days reduced hyperglycemia in diabetic rats through its impact on fasting blood glucose, hemoglobin A1C, and insulin^11^. Jung et al.^12^ reported that hesperidin-treated male C57BL/KsJ-db/db mice had significant reduction in blood glucose compared to control after 5 weeks of intervention. The mechanism of action was suggested through changes in hepatic glucose-regulating/transporting enzyme gene expression, including increase in glucokinase mRNA and decrease in glucose-transporter 2 (GLUT2), which resulted in an increase in hepatic glucose utilization and decreased hepatic glucose output^12^. Furthermore, increased adipocyte glucose-transporter 4 (GLUT4) gene expression was observed, which may contribute to increased glucose uptake by the adipose tissue^12^. However, data in humans is limited and warrant follow-up study to understand the contribution of hesperidin and the non-digestible carbohydrate component of OPF.

While the possible impact of fiber and lower postprandial glycemic excursions on development and/or treatment of type 2 diabetes has been widely discussed, further specific research is required to clearly document any specific relationship or impact on development or progression of disease^13,14^. This study demonstrates the impact of enzyme-treated pomace fiber on postprandial glucose concentrations in healthy human subjects. OJ is a nutrient-rich beverage commonly consumed at breakfast and with the addition of enzyme-treated pomace fiber, it could be an interesting optional food source to improve the general public’s total dietary fiber intake. The definition of dietary fiber is changing continuously with new knowledge and regulatory agencies across the world are working to address various issues and challenges. With growing interest in health promoting functional foods, the demand for natural fiber has increased and use of novel technologies for new sources is on the way. Innovative strategies in the food processing industry has led to extracted fibers from various plants and their by-products and available literature suggests that these by-products are used as a part of health-promoting foods^15^. Future research on by-product dietary fibers should evaluate their health-promoting properties using appropriate human clinical trials for their inclusion in food and beverages.
